# Supplementary material for: The Absence of FAIM Leads to a Delay in Dark Adaptation and Hampers Arrestin-1 Translocation upon Light Reception in the Retina
Source: Cells. 2023 Feb 2;12(3):487. doi: 10.3390/cells12030487 (PMC9914070; doi:10.3390/cells12030487)
Supplement: Supplementary file 1 [file cells-12-00487-s001.zip › cells-2127192-supplementary.pdf]

Supplementary Materials for:

The Absence of FAIM Leads to a Delay in Dark Adaptation and Hampers Arrestin-1 Translocation upon Light Reception in the Retina

Anna Sirés [1,2,3,†], Mateo Pazo-González [4,5,†], Joaquín López-Soriano [1,2,3], Ana Méndez [6,7,8], Enrique J. de la Rosa [4,9], Pedro de la Villa [4,5], Joan X. Comella [1,2,3], Catalina Hernández-Sánchez [4,9] and Montse Solé [1,2,3,*]

1 Cell Signaling and Apoptosis Group, Vall d'Hebron Institute of Research (VHIR), 08035 Barcelona, Spain
2 Centro de Investigación Biomédica en Red sobre Enfermedades Neurodegenerativas (CIBERNED), ISCIII, 28029 Madrid, Spain
3 Departament de Bioquímica i Biologia Molecular i Institut de Neurociències, Facultat de Medicina, Universitat Autònoma de Barcelona (UAB), 08193 Bellaterra, Spain
4 Department of Molecular Biomedicine, Centro de Investigaciones Biológicas Margarita Salas (CSIC), E-28040 Madrid, Spain
5 Department of Systems Biology, Facultad de Medicina, Universidad de Alcalá, 28871 Alcalá de Henares, Spain
6 Department of Physiological Sciences, School of Medicine, Campus Universitari de Bellvitge, University of Barcelona, 08907 Barcelona, Spain; mendezzu@idibell.cat
7 Institut de Neurociències, Campus Universitari de Bellvitge, University of Barcelona, 08907 Barcelona, Spain
8 Institut d'Investigació Biomèdica de Bellvitge (IDIBELL), Campus Universitari de Bellvitge, University of Barcelona, 08907 Barcelona, Spain
9 Centro de Investigación Biomédica en Red de Diabetes y Enfermedades Metabólicas Asociadas (CIBERDEM), ISCIII, 28029 Madrid, Spain

* Correspondence: montserrat.sole@uab.cat
† These authors contributed equally to this work.

Table S1. Table of primary antibodies for immunofluorescence detection.

Antibody	*Supplier information*	*Immunogen*	*Dilution*
Multi-ubiquitin	Santa Cruz Biotechnology mouse monoclonal antibody Cat# sc-8017	UBB human, mouse, rat	1:2,000
GFAP	Abcam rabbit polyclonal Cat #ab7260	Full-length human recom-binant GFAP expressed in bacteria and highly purified	1:2,000
Arrestin-1	Rabbit polyclonal antibody provided by Dr. Ana Méndez	C10C10 epitope (residues 290-297 of bovine arrestin)	1:1,000
Transducin-α	Rabbit polyclonal antibody, provided by Dr. Ana Méndez	Residues 85–103	1:2,000

Table S2. Table of secondary antibodies for immunofluorescence detection.

Antibody	*Supplier information*	*Immunogen*	*Dilution*
Goat Anti-Rabbit IgG (H+L) Antibody, Alexa Fluor 488 Conjugated	Molecular Probes rabbit polyclonal Cat# A-11008	Rabbit IgG (H+L)	1:600
Goat Anti-Rabbit IgG (H+L) Antibody, Alexa Fluor 594 Conjugated	Molecular Probes rabbit Cat# A-11012	Rabbit IgG (H+L)	1:600

Table S3. Table of primary antibodies for western blot.

Antibody	*Supplier information*	*Immunogen*	*MW (kDa)*	*Dilution*
Anti-FAIM	In-house rabbit polyclonal antibody [5]	Recombinant protein corresponding to full-length rat FAIM	21, 23	1:2,000
Arrestin-1	Rabbit polyclonal antibody provided by Dr. Ana Méndez	C10C10 epitope (residues 290-297 of bovine arrestin)	48	1:10,000
Transducin-α	Rabbit polyclonal antibody, provided by Dr. Ana Méndez	Residues 85–103 of transducin-α	40	1:5,000
Anti-α-tubulin	Sigma-Aldrich mouse monoclonal antibody Cat# T5168	B612 clone hybridoma produced by the fusion of mouse myeloma cells and splenocytes from an immunized mouse	50	1:60,000

Table S4. Table of secondary antibodies for western blot.

Antibody	*Supplier information*	*Immunogen*	*Dilution*
Goat anti-Rabbit IgG	Sigma-Aldrich goat Cat# A0545	Purified rabbit IgG	1:20,000
Rabbit anti-mouse IgG	Sigma-Aldrich rabbit polyclonal antibody Cat# A9044	Purified mouse IgG	1:20,000

Figure S1.

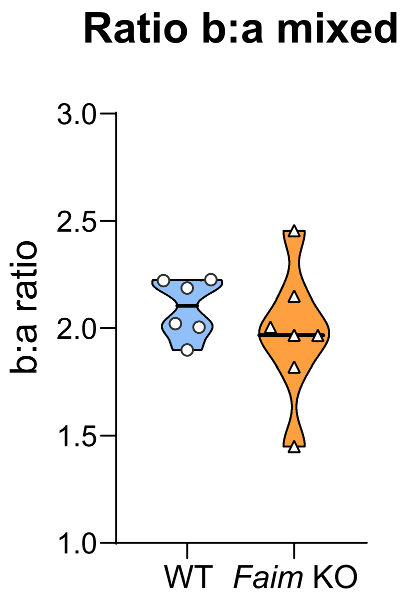

Figure S1. **b:a ratio is normal in Faim KO mice at 18 months.** Ratio of mixed b-and a-wave at 1.5 log cd·s/m². Data are represented as violin plots. Each dot corresponds to a retina, the median is represented by a thick dashed line, and quartiles are represented by thin dotted lines Student's t test statistics, p = 0.3949.

Figure S2.

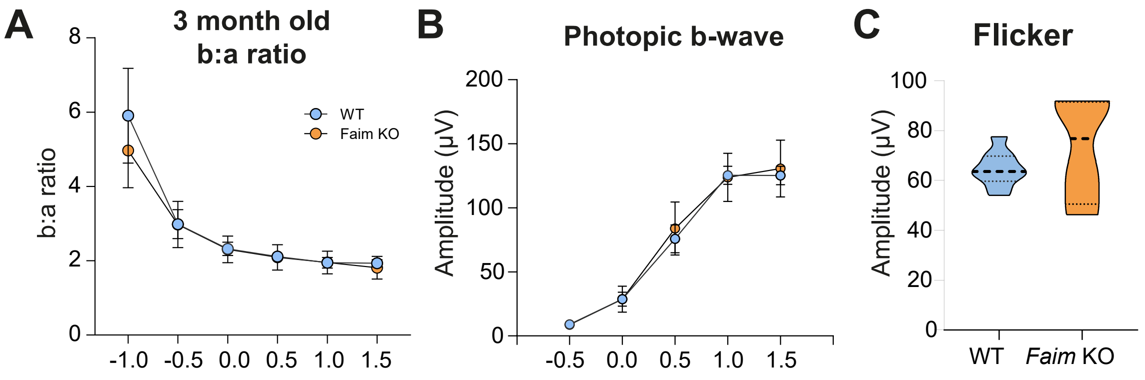

Figure S2. ERG data of WT and Faim KO mice at 3 months of age (A) Mixed b:a ratio is normal in Faim KO mice at 3 months of age at 1.5 log cd·s/m². **(B)** Amplitudes of ERG recordings in photopic conditions under different light intensities. **(C)** Flicker ERG amplitude at 20 Hz. Data are represented as XY graphs and violin plots.

Figure S3.

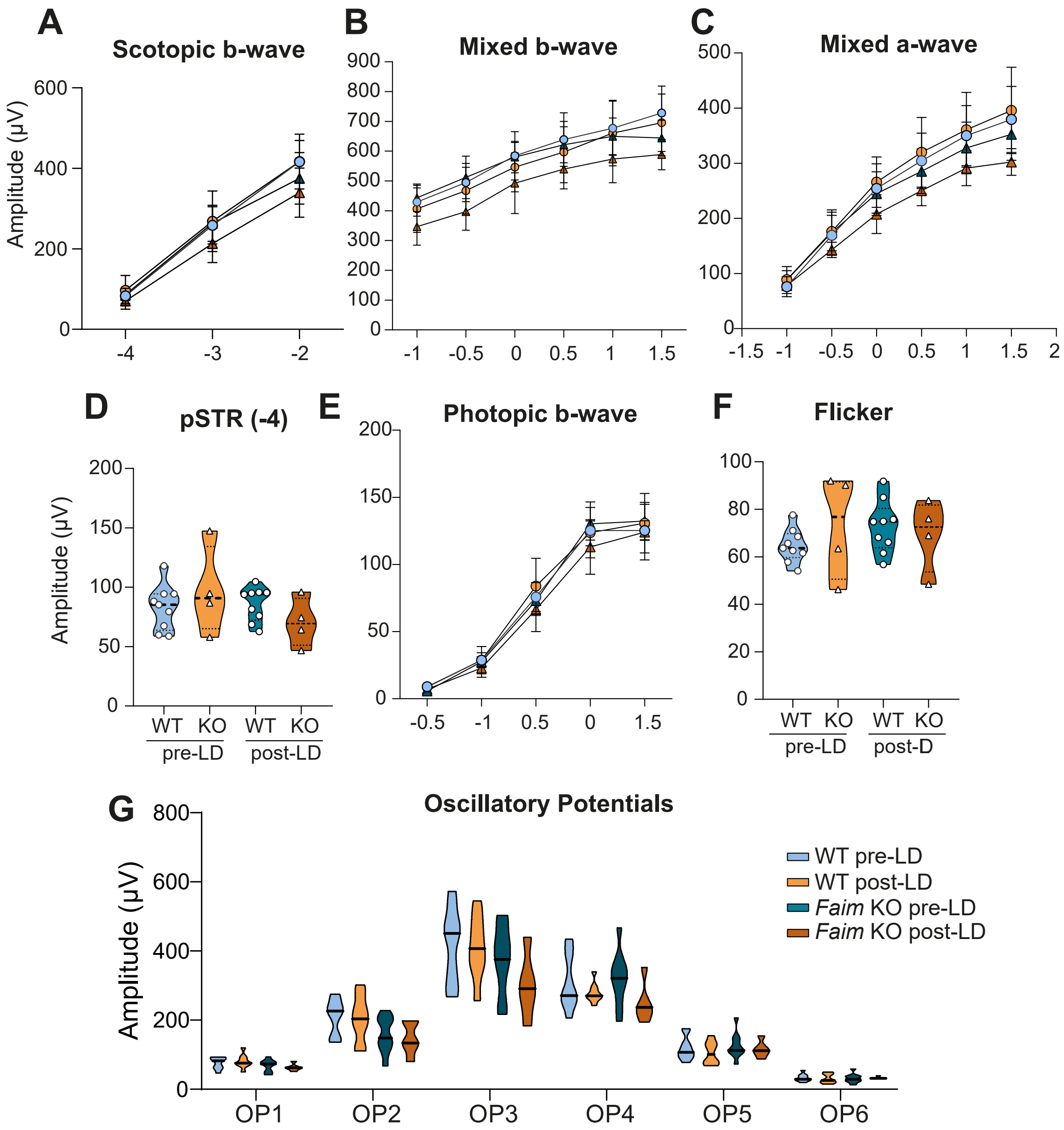

Figure S3. No changes were found in ERG analysis in 2-month-old Faim KO mice after light damage. ERG was performed at the indicated light intensities, corresponding to scotopic **(A),** mixed **(B, C)** and photopic **(E)** conditions. pSTR was performed at -4 log cd·s/m^2 **(D),** and flicker recordings at 20 Hz **(F)**. Oscillatory potential amplitudes are represented in **(G).** At least 4 animals were analysed per each group. Data are represented as XY graphs or violin plots. In violin plots, each animal is represented as a dot. Each violin plot extends from the min to max values, the median is represented by a thick dashed line, and quartiles are represented by thin dotted lines. Statistical analysis was performed using repeated measures three-way ANOVA, in which genotype, light intensity and light damage were used as factors. * $p < 0.05$.
